# The potential of an artificial intelligence for diagnosing MRI images in rectal cancer: multicenter collaborative trial

**DOI:** 10.1007/s00535-024-02133-8

**Published:** 2024-07-31

**Authors:** Atsushi Hamabe, Ichiro Takemasa, Masayuki Ishii, Koichi Okuya, Koya Hida, Daisuke Nishizaki, Atsuhiko Sumii, Shigeki Arizono, Shigeshi Kohno, Koji Tokunaga, Hirotsugu Nakai, Yoshiharu Sakai, Masahiko Watanabe

**Affiliations:** 1 Department of Surgery, Surgical Oncology and Science, Sapporo Medical University, S1 W16, Chuo-Ku, Sapporo, 060-8543 Japan; 2 Department of Gastroenterological Surgery, Graduate School of Medicine, Osaka University, 2-2-E2 Yamadaoka, Suita, Osaka 565-0871 Japan; 3 Department of Surgery, Kyoto University Graduate School of Medicine, Kyoto, Japan; 4 Department of Diagnostic Radiology, Kobe City Medical Center General Hospital, Kobe, Japan; 5 Department of Diagnostic Radiology, Japanese Red Cross Wakayama Medical Center, Wakayama, Japan; 6 Department of Radiology, Mayo Clinic, Rochester, MN USA; 7 Department of Surgery, Osaka Red-Cross Hospital, Osaka, Japan; 8 Department of Surgery, Kitasato University Kitasato Institute Hospital, Tokyo, Japan

**Keywords:** Rectal cancer, MRI, Artificial intelligence

## Abstract

**Background:**

An artificial intelligence-based algorithm we developed, mrAI, satisfactorily segmented the rectal tumor, rectum, and mesorectum from MRI data of rectal cancer patients in an initial study. Herein, we aimed to validate mrAI using an independent dataset.

**Methods:**

We utilized MRI images collected in another nationwide research project, "Open versus Laparoscopic Surgery for Advanced Low Rectal Cancer Patients". MRIs from 467 cases with upfront surgery were utilized; six radiologists centralized the MRI evaluations. The diagnostic accuracies of mrAI and the radiologists for tumor depth were compared using pathologic diagnosis as a reference.

**Results:**

For all cases, centralized diagnosis demonstrated 84.2% sensitivity, 37.7% specificity, and 73.7% accuracy; mrAI exhibited 70.6% sensitivity, 61.3% specificity, and 68.5% accuracy. After limiting MRIs to those acquired by a Philips scanner, with an inter-slice spacing of ≤ 6 mm—both conditions similar to those used in the development of mrAI—the performance of mrAI improved to 76.8% sensitivity, 76.7% specificity, and 76.7% accuracy, while the centralized diagnosis showed 81.8% sensitivity, 36.7% specificity, and 71.3% accuracy. Regarding relapse-free survival, the prognosis for tumors staged ≥ T3 was significantly worse than for tumors staged ≤ T2 (*P* = 0.0484) in the pathologic diagnosis. While no significant difference was observed between ≥ T3 and ≤ T2 tumors in the centralized diagnosis (*P* = 0.1510), the prognosis for ≥ T3 was significantly worse in the mrAI diagnosis (*P* = 0.0318).

**Conclusion:**

Proper imaging conditions for MRI can enhance the accuracy of mrAI, which has the potential to provide feedback to radiologists without overestimating tumor stage.

## Introduction

In the treatment of locally advanced rectal cancer, neoadjuvant chemoradiotherapy (CRT) has long been a standard of care in Western countries. More recently, total neoadjuvant therapy (TNT) has emerged as an intensified option proven to reduce the risk of distant metastasis [1–3]. However, there are patients who can achieve a cure without neoadjuvant treatment, for whom neoadjuvant CRT or TNT might be harmful [4, 5]. As multimodal therapies grow increasingly complex, accurate diagnosis of baseline tumor characteristics becomes vital for individualized decision-making. Among the preoperative assessments, MRI stands out as the most crucial tool, capable of revealing the tumor's various malignant features [6, 7]. Yet, even when interpreted by expert radiologists, the accuracy of MRI findings can be further refined. The development of technology to support radiologic interpretation of MRI is crucial in improving the prognosis of locally advanced rectal cancer and optimizing its treatment.

In our prior research, we developed an AI-based algorithm to segment the rectal tumor, rectum, and mesorectum from MRI data (referred to as mrAI). This can evaluate the T stage or identify areas at risk of circumferential resection margin (CRM) involvement [8]. While there have been recent developments in AI systems for diagnosing rectal cancer, our mrAI stands out, in that it was created using ground-truth data that aligns cancerous areas in pathologic specimens with corresponding regions on high-resolution MRI. Moreover, it segments three distinct areas—the tumor, rectum, and mesorectum—offering visual insights useful for selecting the optimal dissection layer during surgery and ensuring the preservation of the CRM. Although the precision of this technology was deemed satisfactory, it was developed using a ground-truth label based solely on MRI data from one institution (Sapporo Medical University) with a single data acquisition protocol. Consequently, additional validation using an independent dataset is essential to highlight the utility of mrAI.

The Japanese nationwide study titled “"Open versus laparoscopic surgery for advanced low rectal cancer patients” was conducted across 69 institutes. This research was a project under the Japan Society of Laparoscopic Colorectal Surgery (JSLCS) spanning the period from January 2010 through December 2011 [9]. In a subsequent survey, MRI data were retrospectively collected to analyze correlations between MRI-related factors and clinicopathological outcomes. Here, MRIs underwent centralized review by expert radiologists. This study highlighted the result that lateral pelvic lymph node dissection (LPND) improved recurrence-free survival for cases with a lateral pelvic node short axis ≥ 5 mm [10], and a nomogram was devised to anticipate metastasis to the lateral pelvic node [11]. In the present research, our aim was to validate mrAI using the MRI dataset collected in the JSLCS study.

## Materials and methods

### Study design and participants

This retrospective study utilized data from a multicenter cohort study spanning 69 institutions affiliated with the JSLCS. In the initial study, 1608 patients diagnosed with clinical stage II/III low rectal cancer below the peritoneal reflection and who underwent rectal resection between January 2010 and December 2011 were registered [9]. Clinical data were prospectively collected, demonstrating that laparoscopic surgery could be a feasible treatment option for advanced low rectal cancer (UMIN registration number: 000013919). In a subsequent study, 752 MRIs were retrospectively analyzed to identify the subset of patients who could benefit from LPND [10]. Six radiologists reviewed the MRI-related findings, such as tumor depth, lymph node enlargement, mrEMVI [12, 13], and mrCRM [14], revealing that LPND was advantageous for patients with a lateral pelvic node measuring between 5 and 10 mm. The study received approval from the institutional review boards of Kyoto University and all participating centers (UMIN registration number: 000026789). An opt-out method was used to obtain consent for study inclusion, as well as potential secondary data usage, in line with the Japanese Ethical Guidelines for Medical and Health Research Involving Human Subjects. All procedures performed in studies involving human participants were in accordance with the ethical standards of the institutional and/or national research committee and with the 1964 Declaration of Helsinki and its later amendments or comparable ethical standards. In the current study, the validity of mrAI was assessed using these datasets after securing approval from the institutional review boards of Sapporo Medical University and Kyoto University. A total of 503 patients who did not receive preoperative treatment were selected to compare the mrAI-predicted T stage and the radiologic T stage diagnosed by the certified radiologists. The pathologic stage was used as a reference.

### Interpretation of MRI

**Fig. 1:**
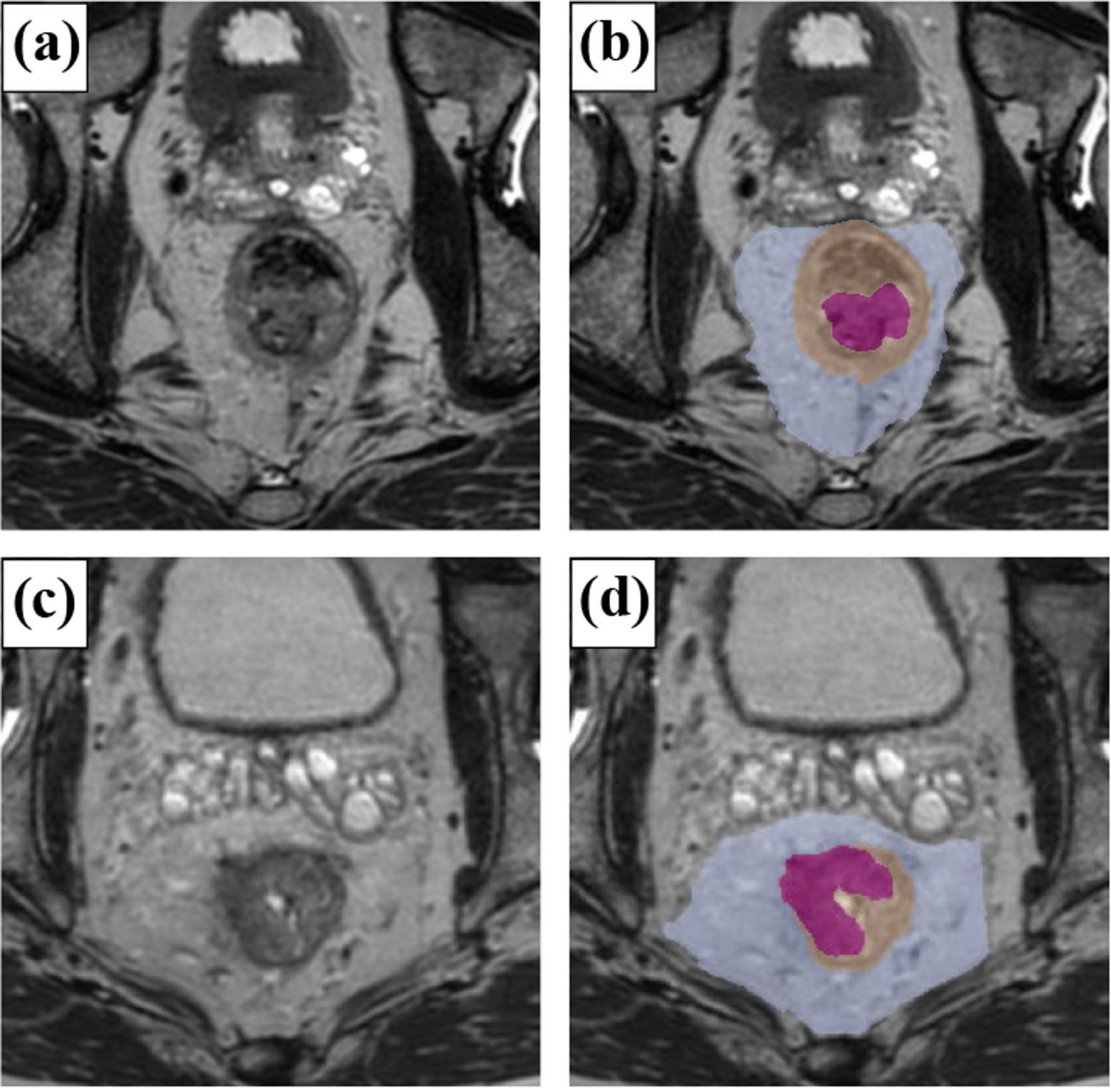
Segmentation images based on mrAI. **a** MRI image of rectal cancer diagnosed as T2 by mrAI. **b** Identical case as (**a**) overlaid with segmentation results. Pink area indicates tumor, beige area indicates rectum, and blue area indicates mesorectum. **c** MRI image of rectal cancer diagnosed as T3 by mrAI. **d** Identical case as (**c**) overlaid with segmentation results. Pink area indicates tumor, beige area indicates rectum, and blue area indicates mesorectum

As detailed in a previous study, six radiologists, after a consensus meeting on MRI findings, reviewed the MRIs to assess tumor depth, mrCRM, and lymph-node size [10]. Tumor depth was categorized as T0–2, T3, or T4. It was classified as T3 if the tumor penetrated the muscularis propria and as T4 if it invaded adjacent organs. A detailed description of mrAI is available in a prior report [8]. In summary, the algorithm was developed using a deep neural network. MRIs were annotated with ground-truth labels of tumors by verifying pathologically confirmed lesions on sections of circular resected specimens. The T stage was semi-automatically determined based on the positional relationships among the tumor, rectum, and mesorectum. Radiologists and mrAI evaluated the T2-weighted axial images of the collected MRIs. MRI data were loaded onto a laptop equipped with mrAI for analysis. The algorithm generated segmentation results for the tumor, rectum, and mesorectum (Fig. 1), from which the T stage and mesorectal fascia (MRF) involvement were automatically calculated. We categorized the MRI diagnosis into three groups, in which “local diagnosis” was the diagnosis made at each hospital, “centralized diagnosis” was made by the above qualified radiologists, and “mrAI diagnosis” was made by mrAI.

### Statistical analyses

All statistical analyses were conducted using JMP Pro 16 (SAS Institute, Cary, NC). Results are displayed as either the number of cases evaluated (for categorical data) or the median and range (for quantitative data). Univariate analyses utilized Fisher’s exact test. The Kaplan–Meier method estimated relapse-free survival, and the log-rank test determined statistical significance. All P values were two-tailed, with *P* values < 0.05 deemed statistically significant. A 2 × 2 table was used to calculate the sensitivity, specificity, and accuracy of MRI. For example, in calculating the characteristics of the diagnostic test for ≥ T3 staging, sensitivity is defined as the proportion of cases staged as ≥ T3 on MRI among pathologic ≥ T3 cases, specificity is the proportion of cases staged as ≤ T2 on MRI among pathologic ≤ T2 cases, and accuracy is defined as the proportion of cases in which the pathologic tumor depth (≤ T2 or ≥ T3) was correctly diagnosed by MRI in all evaluated cases.

## Results

### Patients

**Fig. 2:**
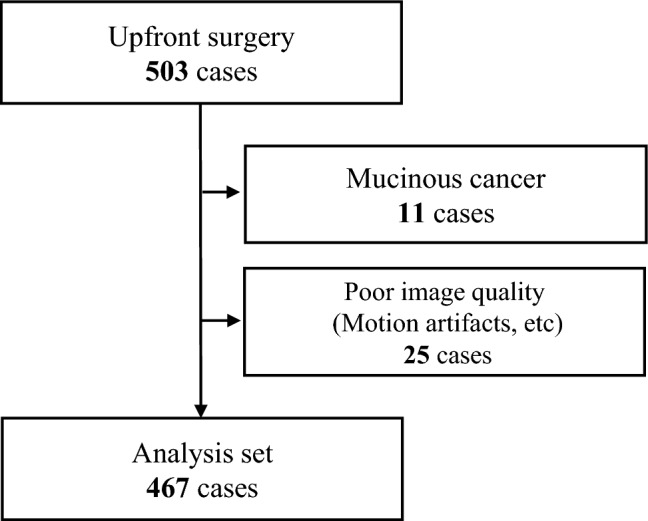
Flowchart of patient inclusion in the study

From the enrolled cases, 11 with mucinous carcinoma were excluded because they were demonstrated to be unsuitable for analysis by mrAI due to characteristic MRI findings. In addition, 25 cases were excluded due to poor MRI quality, such as motion artifacts. Consequently, 467 cases were included in our analysis (Fig. 2).

The median duration of post-surgical follow-up was 2119 days (interquartile range: 1620–2418 days). Table 1 presents the patients' characteristics, MRI details, TNM classification, surgical procedures performed, and pathologic findings. The median age of the patients was 63 years (range: 26–86 years), and 317 of the 467 patients were male.

**Table 1:** Patients’ characteristics, MRI details, TNM classification, surgical procedures performed, and pathologic findings

Patient characteristics	
Age in years, median (range)	63 (26–86)
Biologic sex, male/female	317/150
BMI (kg/m2), median (range)	22.3 (15.0–40.9)
PS, 1/2/3/4	174/263/28/1
MRI	
Magnetic field strength (T), 1.0/1.5/3.0/unknown	1/349/92/25
In-plane resolution (mm), median (range)	0.52 × 0.52 (0.27 × 0.27–1.02 × 1.02)
Inter-slice spacing (mm), median (range)	6.1 (3.0–10)
Manufacture, P/G/S/T/H^a^	242/106/90/25/4
Tumor characteristics at initial examination at each hospital	
Distance from anal verge (cm), median (range)	5 (0–12)
cT1/2/3/4	3/33/351/80
cN, negative/positive/unknown	173/292/2
Suspected metastasis to lateral lymph nodes	59
Operative results	
Approach, open/laparoscopy	329/138
Procedure, anterior resection/ISR/APR/Hartmann/extended resection	233/79/123/15/17
LPND, yes/no	281/186
Pathologic findings	
Histology, tub1/tub2/pap/por/sig	146/299/6/15/1
pT1/2/3/4	11/95/309/52
pN0/1/2/3	228/123/68/48
pM0/1	462/5
pStage I/II/IIIa/IIIb/IV	66/162/122/112/5
Metastasis to lateral lymph node, yes/no	43/424

APR, abdominoperineal resection; BMI, body mass index; IQR, interquartile range; ISR, intersphincteric resection; LPND, lateral pelvic lymph node dissection; pap, papillary adenocarcinoma; por, poorly differentiated adenocarcinoma; PS, performance status.; sig, signet-ring cell carcinoma; tub1, well-differentiated adenocarcinoma; tub2, moderately-differentiated adenocarcinoma

^a^P, Philips Medical Systems; G; GE Medical Systems; S, SIEMENS; T, Toshiba; H, Hitachi Medical Corporation

MRI images were primarily obtained using 1.5-T MRI scanners (*N* = 349). The median inter-slice spacing was 6.1 mm (range: 3.0–10 mm), and the median in-plane resolution was 0.52 × 0.52 mm. The manufacturers of the MRI scanners included Philips (242 cases), GE Healthcare (106 cases), Siemens (90 cases), and others (29 cases). The relationship between manufacturers and magnetic field strength is shown in Supplementary Table 1.

Table 1 also displays the clinical T and N staging determined at each hospital. There were 431 cases staged as cT3/4. The median distance from the anal verge was 5 cm. Laparoscopic surgeries were performed in 138 cases, and the procedures included 233 anterior resections. LPND was performed in 281 cases.

Pathologic findings revealed that there were 106 cases of pT1/2, which was more frequent than the local diagnosis which was made at each hospital (*P* < 0.0001).

### Precision of T staging

Following the method adopted in the previous studies that examined the performance of MRI diagnosis for rectal cancer [15–19], we initially assessed the diagnostic performance of mrAI for T staging, comparing it with that of radiologic experts (Table 2). For all cases, centralized diagnosis had an 84.2% sensitivity, 37.7% specificity, and 73.7% accuracy, whereas mrAI diagnosis exhibited a performance of 70.6% sensitivity, 61.3% specificity, and 68.5% accuracy. Local diagnosis showed 96.1% sensitivity, 20.8% specificity, and 79.0% accuracy. Given that the entire set comprised various MRIs acquired under different protocols or with different scanners, we hypothesized that the suboptimal results from mrAI might be due to discrepancies in image quality between the analyzed MRIs and those used to develop the algorithm. As noted in our previous report [8], we used high-resolution or 3D MRIs obtained with a Philips scanner. Thus, we evaluated the performance of mrAI using MRIs acquired under similar conditions. Out of all cases, 226 had an inter-slice spacing of 6 mm or less. Analysis of these cases indicated that mrAI diagnostic performance improved to 73.0% sensitivity, 67.3% specificity, and 71.7% accuracy, while the performance based on centralized diagnosis as well as local diagnosis remained similar to that in the first evaluation. Subsequently, MRIs acquired with a Philips scanner were isolated, and 129 cases were analyzed (selected group). The performance of mrAI furthermore improved to 76.8% sensitivity, 76.7% specificity, and 76.7% accuracy, while the performance of the centralized diagnosis was consistent with that in the first analysis. Local diagnosis yielded a performance of 99.0% sensitivity, 23.3% specificity, and 81.4% accuracy. These data suggested a tendency for local diagnosis to overestimate the tumor stage. As for diagnosis by mrAI, scanners from the other manufacturers did not yield results as favorable as those from Philips.

**Table 2:**
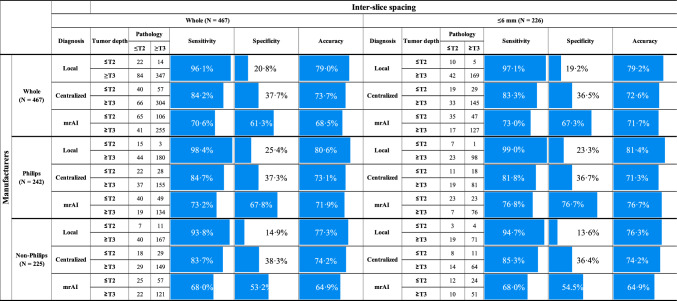
Comparison of the diagnostic accuracy of the local, centralized, and mrAI diagnoses for ≤ T2 and ≥ T3, using pathological diagnosis as a reference

The diagnostic performance for stage T4 tumors was further detailed by categorizing tumor depth as ≤ T2, T3, or T4 in the selected group. In the diagnosis of T4 tumors, the sensitivity and specificity were 85.7%/90.4% for local diagnosis, 57.1%/94.8% for centralized diagnosis, and 28.6%/97.4% for mrAI-based diagnosis (Supplementary Table 2).

### Precision of predicting MRF involvement

We aimed to validate the accuracy of mrAI in predicting MRF involvement. However, we identified two major challenges in verifying the presence of MRF involvement by pathology. First, at the time the analyzed cases underwent surgery, the CRM was not generally evaluated using a circumferential specimen in Japan. Second, even if MRF involvement was present, the resection of surrounding tissues such as nerves might result in the absence of cancer infiltration at the resection margin of the specimen. Therefore, we decided to evaluate the performance of mrAI by assessing the correlation of MRF involvement between centralized diagnosis and mrAI diagnosis. As shown in Table 3, in the selected group, the mrAI evaluation significantly correlated with the centralized diagnosis (*P* = 0.0003).

**Table 3:** The correlation of mesorectal fascia involvement between centralized diagnosis and mrAI diagnosis

	Centralized diagnosis	
	Negative	Positive
mrAI diagnosis		
Negative	77	35
Positive	4	13

### Long-term survival

**Fig. 3:**
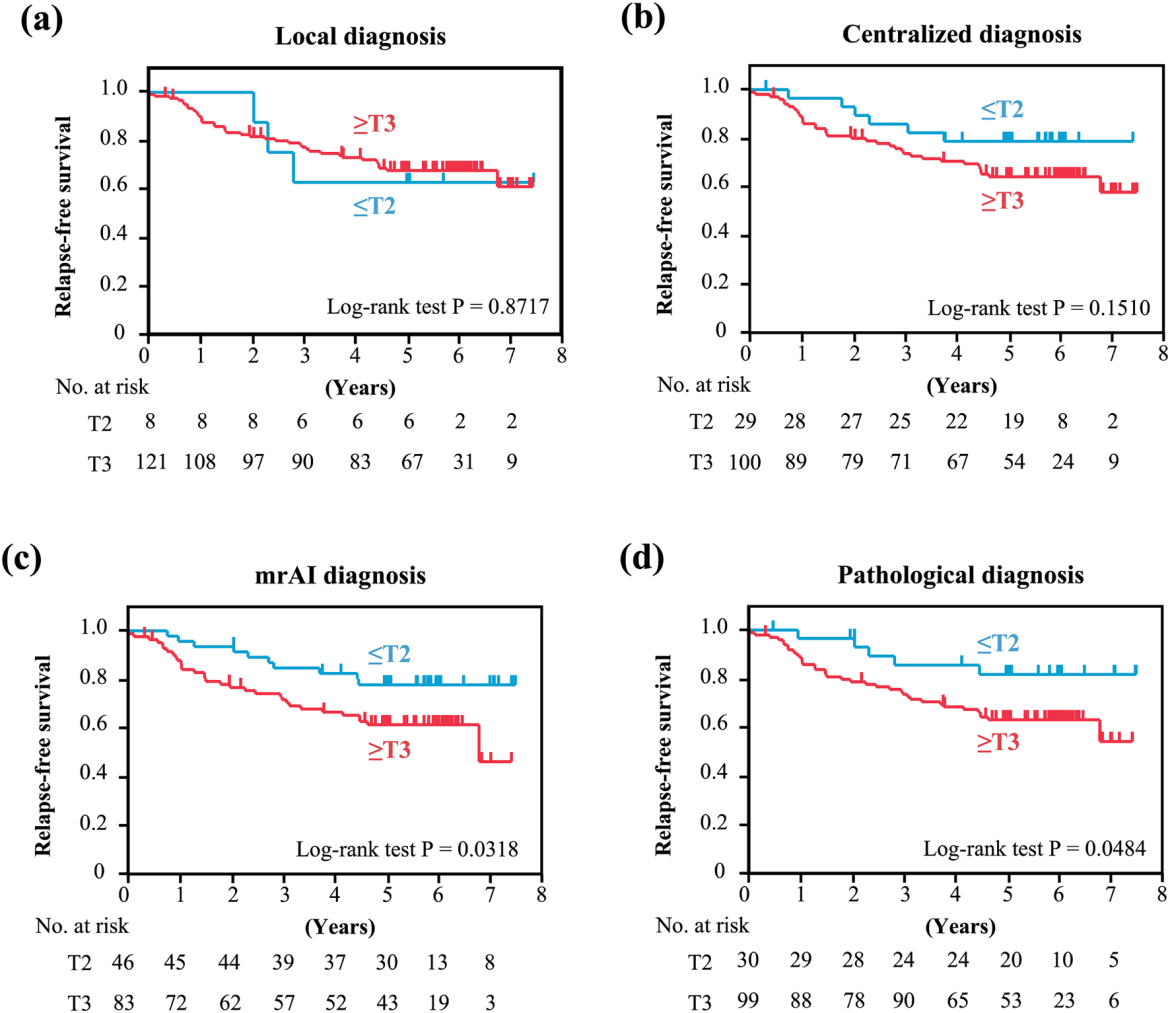
Relapse-free survival based on depth classification per diagnostic method. **a** Local diagnosis. **b** Centralized diagnosis. **c** mrAI diagnosis. **d** Pathologic diagnosis

Regarding relapse-free survival, we evaluated the association with long-term prognosis for each diagnostic modality by comparing ≥ T3 with ≤ T2 tumors. In local diagnosis, no significant difference was observed between ≥ T3 and ≤ T2 (*P* = 0.8717) (Fig. 3a). While no significant difference was observed between ≥ T3 and ≤ T2 in the centralized diagnosis (*P* = 0.1510), the prognosis for ≥ T3 was significantly worse in the mrAI diagnosis than it was for ≤ T2 (*P* = 0.0318), consistent with the pathologic results in which the prognosis for ≥ T3 was significantly worse compared with that for ≤ T2 (*P* = 0.0484) (Fig. 3b–d).

## Discussion

In this study, we were able to verify the usefulness of mrAI, which we developed using a multi-institutional dataset. The performance of mrAI was enhanced when characteristics of the MRI imaging environment, such as the imaging protocol and the manufacturer, were close to those of the ground-truth data used for algorithm development. This result suggests how sensitive AI can be as a diagnostic support technology and has revealed that addressing this issue will be mandatory in making this technology generally available in future. The result that mrAI had higher diagnostic accuracy with thinner slice thickness suggests that image quality improvement of each cross-sectional image and the information embedded within the continuity of multiple sections are likely important in enhancing diagnostic accuracy.

The most standard approach for assessing the accuracy of preoperative diagnosis is comparison with pathologic findings. While such verification was previously possible, it has become challenging to use unmodified specimens now that preoperative treatment has been standardized [15–18]. Japan has a unique environment where unmodified pathologic information from relatively recent times is easily accessible, as surgery-first treatment has been standardized for a long time, even after neoadjuvant CRT became prevalent in the West. In this study, to develop an AI-based algorithm, we created ground-truth label data by correlating pathologic sections of circular specimens with MRI images. To assess diagnostic accuracy, we utilized nationwide, multicentric data to compare pathologic findings with MRI on a 1:1 basis. In Japan too, the importance of preoperative treatment has been recognized in recent years, and the number of cases undergoing preoperative treatment is increasing [20, 21]. Conducting similar research methods will become more and more challenging in future. We consider this study to have significant value for having utilized this rare opportunity.

Regarding T staging, MRI is perceived to have a tendency to overestimate tumor stage rather than underestimate it [22]. In the MERCURY study, a comparison was made between MRI diagnosis and pathologic diagnosis among 311 individuals who underwent surgery first. Of the cases pathologically diagnosed as T1/2, 36% were diagnosed as T3/4 on MRI, while 31% of the cases pathologically diagnosed as T3/4 were diagnosed as T1/2 on MRI [15]. Long-term prognosis analysis of the MERCURY study showed a poor disease-free survival rate in cases with a high risk of positive CRM on preoperative MRI. In this report, 23.2% were diagnosed as Stage I on preoperative MRI, and eventually 26.7% were diagnosed as Stage I pathologically. This fact suggests that among the cases diagnosed pathologically as T2 or lower, there were not a few cases diagnosed as T3/4 at the time of diagnosis [23]. Although the MERCURY study had strict management with tightened MRI diagnostic criteria, it is believed that the tendency to overestimate the depth in actual clinical settings may be even more pronounced. Other retrospective studies examining the diagnostic accuracy of MRI for upfront surgery cases have also shown a tendency for MRI diagnosis to overestimate tumor stage [16–18], and a meta-analysis analyzing MRI diagnostic accuracy reported that the proportion of overestimation was 25% and of underestimation was 13% [19]. In not a few cases, it is difficult to distinguish between fibrotic and tumor tissues [22]. In radiologic diagnosis made by humans, intentions to avoid under-diagnosis by considering various clinical situations may significantly influence the diagnosis. In our study data, a similar trend was observed. Particularly in this study, which analyzed data from a time when MRI was not widespread in Japan, there was a tendency to extremely over-diagnose in real-world clinical settings. In centralized diagnosis involving experienced radiologists, this tendency was corrected compared with local diagnosis, but 63% of the cases pathologically diagnosed as T2 or lower were over-diagnosed preoperatively as T3 or higher. While a central diagnostic setting is more conducive to neutral judgments, it would be even harder to avoid over-diagnosis in real-world clinical settings [24]. In this regard, mrAI's diagnosis is always neutral, and even in cases that are difficult to judge, the algorithm can diagnose tumors without bias as T2 or below. This may contribute to optimizing each patient's treatment. We do not assert that mrAI surpasses human diagnostic capabilities; rather, we emphasize that AI diagnosis should not be seen as a technology that confronts radiologic diagnosis. A comprehensive understanding of both the advantages and disadvantages of mrAI is imperative, and its application should be judiciously implemented to augment the diagnostic procedures conducted by radiologists. In addition, mrAI, when applied to research using MRI, is expected to be able to make unbiased decisions and also have the potential to reduce the workload of radiologists performing central diagnoses, since they can carry out bias-free diagnostics.

In this study, to evaluate the performance of mrAI, the depth of invasion was extracted based on segmentation and compared with centralized diagnosis. The depth of invasion is a well-known long-term prognostic factor, and in the MERCURY study the hazard ratio for DFS of pathologic Stage II was over five times that of Stage I [23]. Compared with centralized diagnosis, mrAI showed slightly lower sensitivity and higher specificity. To validate the clinical significance of this result, we compared the relapse-free survival of ≤ T2 and T3 ≤ groups in MRI diagnosis. Patients with pathologic stage ≥ T3 tumors had significantly poorer prognosis than those with tumors staged ≤ T2, but a significant difference in relapse-free survival was shown only in the diagnosis derived by mrAI, not in the centralized diagnosis. Moreover, no association was found between the two groups in local diagnosis. Although we could not evaluate the performance of mrAI against pathologic assessment for MRF involvement, the observed correlation between centralized diagnosis and mrAI diagnosis suggests that mrAI may be useful for assessing MRF involvement.

An essential factor in deciding on preoperative treatment for locally advanced rectal cancer is the risk of recurrence for each case. The recently highlighted TNT has been proven to improve DFS in locally advanced rectal cancer, and its application is expanding, especially in Western countries [1–3]. However, for cases that can be cured without TNT, it represents overtreatment. One of the current challenges is to clearly define the indications for TNT [25]. We expect that mrAI, which can classify relapse-free survival similarly to pathologic diagnosis based on MRI findings at the time of diagnosis, will play a significant role in rectal cancer treatment as multidisciplinary treatments evolve. Especially as mentioned above, there might be a concern among radiologists about missing the opportunity for preoperative treatment, which tends to lead to overestimation. The importance of a tool to support neutral judgment is immense. While MRI can evaluate factors such as extramural vascular invasion (EMVI) [26], lymph node metastasis [27], and tumor deposits [28] that also affect long-term prognosis, they are not assessed by the current mrAI. We have not explored these in this study but plan to do so in future research. Furthermore, with recent advances in ctDNA and multiomics analysis, individualization of colorectal cancer treatment is becoming a reality [29–32]. With these technological innovations, establishing a method to comprehensively evaluate the individual risks of advanced rectal cancer will help pave the way to future individualized treatment of rectal cancer.

There are several limitations to this study. First, this study was retrospective, and therefore there is potential for bias. Because the MRIs were not obtained within a recent timeframe, in some cases the image quality was inferior compared with that of MRI obtained with standardized imaging protocols in Western countries [22, 33]. Second, our findings could not prove that the current mrAI is universally applicable across different vendors and imaging environments. However, optimizing the imaging environment for MRI can enhance the applicability of mrAI, and we believe this challenge can be adequately addressed in future. Third, we did not individually review the segmentation results extracted by mrAI for each case. The accuracy of these segmentation results needs to be prospectively accumulated and verified, ensuring proper imaging conditions.

In conclusion, we were able to verify the performance of mrAI, which we developed using multi-institutional data. By ensuring proper imaging conditions for MRI, the accuracy of the results of the mrAI analysis can be enhanced, and mrAI has the potential to provide feedback to radiologists without leading to overestimation of tumor stage.

## Abbreviations

AI: Artificial intelligence
CRM: Circumferential resection margin
CRT: Chemoradiotherapy
DNA: Deoxyribonucleic acid
EMVI: Extramural vascular invasion
JSLCS: Japan Society of Laparoscopic Colorectal Surgery
LPND: Lateral pelvic lymph node dissection
MRI: Magnetic resonance imaging
TNT: Total neoadjuvant therapy
